# Theoretical and Experimental Studies on Thermal Properties of Polyester Nonwoven Fibrous Material

**DOI:** 10.3390/ma13122882

**Published:** 2020-06-26

**Authors:** Tao Yang, Xiaoman Xiong, Michal Petrů, Xiaodong Tan, Hiroki Kaneko, Jiří Militký, Atsushi Sakuma

**Affiliations:** 1 Institute for Nanomaterials, Advanced Technologies and Innovation, Technical University of Liberec, 461 17 Liberec, Czech Republic; michal.petru@tul.cz; 2 Department of Material Engineering, Faculty of Textile Engineering, Technical University of Liberec, 46117 Liberec, Czech Republic; xiaoman.xiong@tul.cz (X.X.); xiaodong.tan@tul.cz (X.T.); Jiri.Militky@tul.cz (J.M.); 3 Department of Advanced Fibro-Science, Kyoto Institute of Technology, Matsugasaki, Sakyo-ku, Kyoto 606-8585, Japan; hrbk25@gmail.com (H.K.); sakuma@kit.ac.jp (A.S.)

**Keywords:** polyester, fiber orientation, thermal conductivity, models, modification

## Abstract

Polyester nonwoven fibrous material is widely used in construction and automobile industries for thermal insulation purposes. It is worthy and meaningful to understand the effect of structural parameters on the thermal property. Fiber orientation, as one of the most vital parameters, has a significant effect on thermal property. However, there has been little quantitative analysis focusing on this aspect. This paper theoretically and experimentally analyzes the thermal conductivity of samples with varying fiber orientation. Existing models were selected to predict the thermal conductivity of polyester nonwoven samples. Two different apparatus were applied to carry out the experimental measurements. The relative differences between the predicted and measured results were compared. One commonly used model was modified for accurate prediction. It was shown that some existing models under- or overestimate the thermal conductivity compared to the measured values. The results indicate that the modified model can accurately predict the thermal conductivity of polyester nonwoven materials within a 0.2% relative difference.

## 1. Introduction

Recently, nonwoven fibrous materials have been extensively used in construction and automobile industries due to their high porosity, economical price, lightweight, a large thickness range, good sound absorption, etc. The most common application of nonwoven materials in industries is as a dual insulator (thermal and sound) in buildings [1,2,3]. The thermal property of nonwoven fibrous material has attracted considerable attention. The application in thermal insulation of some bast-fibrous materials such as flax and hemp have been verified due to their suitable insulation properties [4]. However, bast-fibrous materials have a risk for microbial and other contaminants, and their quality should be monitored regularly because of the aging effect. The thermal properties of recycled waste clothing textiles for building application have been reported [5]. The inverse method was adopted to study the thermal properties of a fibrous insulator due to the arbitrary distribution of fiber size in waste clothing textiles. Cerkez et al. [6] presented the thermal insulation property of a multi-component air-laid nonwoven and stated that an increase in the amount of glass fiber resulted in lower thermal insulation. A novel approach used to apply silica aerogel into nonwoven fibrous materials for thermal insulation purpose has also been reported [7]. It was found that an aerogel encapsulated nonwoven composite has a remarkable rise on thermal resistance compared to the sample without aerogel. 

Aside from the influence of material on the thermal properties of a nonwoven fibrous material, the effect of producing technology and structural characteristics needs to be considered. Thickness and porosity have been confirmed as the most important factors to determine the thermal properties of nonwoven fibrous materials [8]. The thermal insulation property of perpendicular-laid and cross-laid high-loft nonwoven materials has been studied [9]. According to the thermal conductivity results, cross-laid nonwoven has better thermal insulation than perpendicular-laid nonwoven. It was also found that the thermal conductivity decreased when the density increased in these two high-loft nonwoven structures. In addition, nonwoven fibrous materials made from coarse fibers have higher thermal resistance when the compression load is applied. 

A number of studies have reported on the effect of material type, structural parameter, and manufacturing technology on the thermal property of nonwoven fibrous materials. However, one important factor that researchers have not treated in much detail is fiber orientation. It has been reported that fiber orientation has a significant influence on thermal properties [10,11,12]. If the fibrous materials have the same fiber components, fiber orientation should be primarily considered. Some existing models can be used to estimate the thermal conductivity of nonwoven material when fibers are randomly, perpendicularly, and parallelly orientated to the direction of heat flow [13]. However, these models do not involve the accurate effect of fiber orientation. 

The main objective of this paper was to study the thermal properties of a polyester nonwoven fibrous material, especially for materials with different through-plane fiber orientations. The rest of this paper is organized as follows. Thermal conductivity models for fibrous material are recalled in Section 2. Section 3 outlines the materials, methods of theoretical study, and experimental study. The results from the theoretical and experimental studies are presented and analyzed, along with modifications on an existing model in Section 4, followed by a brief conclusion. 

## 2. Review of Thermal Conductivity Models

Theoretically, thermal conduction always occurs if a temperature gradient exists between a material system and the environment or inside a material system [14]. It is considered that no natural convection occurs in fibrous material with a density larger than 20 kg/m^3^ because the fibers subdivide the gas into sufficiently small pores [15,16]. Additionally, it has been stated that the convection can be eradicated due to the significant friction that is caused by constituent fibers against natural convection [17]. Sun and Pan [14] stated that heat transfer via radiation can be ignored when the temperature gradient is small. The heat transfer through radiation will be considered when the temperature is higher than 500 K [18]. Furthermore, it has been stated that heat transfer modes are generally dependent on the ambient temperature and fibrous material porosity [19]. Thus, thermal conduction is the dominant mechanism in most situations when heat is transferred through a nonwoven fibrous material.

Most of the models used to analyze the heat transfer behavior of fibrous materials have been developed based on electrical network analysis, which is called thermal-electrical analogy [20,21]. Some models based on thermal-electrical analogy will be introduced, and several semiempirical models are included in this section. 

### 2.1. Semi-Empirical Models

Semi-empirical models are proposed for the quick assessment of the thermal conductivity of fibrous materials. Schuhmeister [22] presented an empirical equation to simply calculate the thermal conductivity of a homogeneous and isotropic nonwoven material in 1877. Baxter [23] modified Schuhmeister’s Equation to estimate the conductivity of a wool fibrous material. A semi-empirical model that applied an empirical coefficient for a different type of fiber was developed by Verschoor and Greebler [24]. Some semi-empirical models for thermal conductivity estimation are summarized in Table 1.

### 2.2. Models Based on Thermal-Electrical Analogy

**Figure 1:**
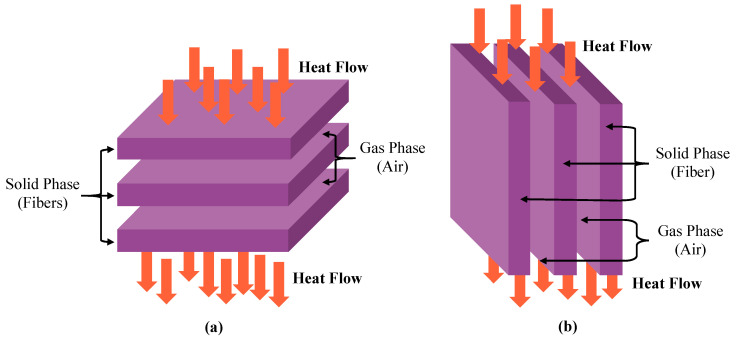
Fibers orientated perpendicular (**a**) or parallel (**b**) to the direction of heat flow.

Electronic components are connected in parallel or series arrangement in an electrical network. Similarly, fibers in a fibrous material are assumed to be perpendicularly (serially), parallelly, or randomly distributed in the direction of heat flow [26]. The sketches of fibers orientated parallel and perpendicular to the direction of heat flow are shown in Figure 1. In parallel arrangement ((b) in Figure 1), the relatively high conductivity of the fibers contributes their maximum effect on the overall conductivity of the fibrous material. In contrast, the fibers contribute a minimum effect to the overall thermal conductivity of the fibrous material because of the air layer between each fiber element in a perpendicular arrangement.

Bogaty et al. [27] proposed models for fibrous material with different fiber arrangements. Bhattacharyya [16] presented two models to predict the thermal conductivity of samples with fibers perpendicularly and randomly to the heat flow based on Fricke’s [21] method for electrical conductivity. Some models used to simply calculate the thermal property of fibrous material with parallel, perpendicular, and random fiber orientations are summarized in Table 2.

These models have been adopted to predict the thermal properties of woven, knitted, and nonwoven fibrous materials [27,28,29]. Militký et al. [28] accurately predicted the thermal resistivity of wool/polyester plain woven fabric by computing the average value of models 5 and 6 in Table 2. Bogaty [27] obtained the relative specific thermal conductivity of wool, cotton, nylon, and orlon fibrous materials by using the calculated values of the effective fraction of fibers orientated parallel and perpendicular to the heat flow via model 8. Nevertheless, these models provide a rough estimation of the thermal resistivity of fibrous material. It has been proven that fiber orientation has a significant influence on thermal conductivity [10,30]. Thus, a more precise prediction that takes into account the specific fiber orientation angle should be considered. The mechanistic assumption that the thermal conductivity in fiber oriented to an arbitrary angle from the heat flow direction is simply the sum of the contributions of conductivities in the fiber axis direction and radial direction used. This assumption is supported by the Debye equation for the prediction of the thermal conductivity [31].

**Figure 2:**
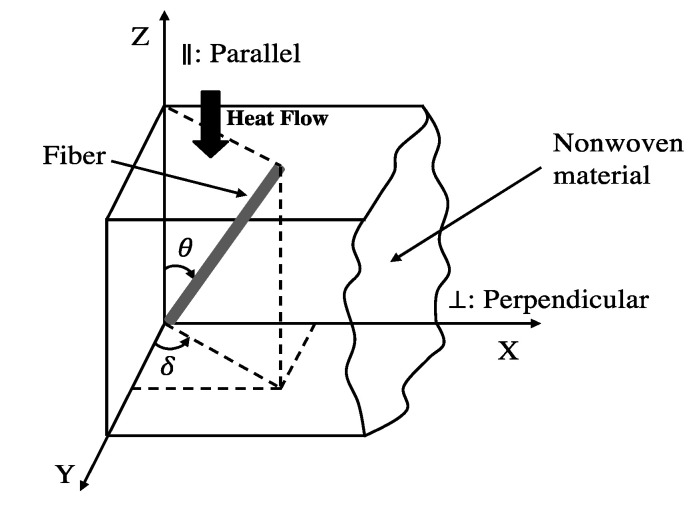
Fiber orientation angle in three-dimensional nonwoven fibrous material.

The fiber orientation in nonwoven fibrous materials refers to the fiber orientation angle, as shown in Figure 2. Angles θ and δ represent the through-plane and in-plane orientation angles, respectively. When θ is zero, the fibers are parallel to the direction of heat flow. If θ is 90°, the fibers are perpendicular to the direction of heat flow. Fibers in the through-plane orientation have a significant effect on thermal properties while the in-plane orientation has less effect [30]. Thus, the angle θ is a critical value in the demonstrated situation where the direction of heat flow is parallel to the Z-axis. 

One series of model that can be used to predict the thermal conductivity of fibrous material with varying fiber orientation based on Bhattacharyya’s [16] research was developed by Stark and Fricke [15] in 1993. They stated that it is necessary to pay attention to the effect of contact between fibers on conductivity. The basic model of thermal conductivity of fibrous material based on Bhattacharyya’s assumptions is represented as:

(1) $$k_{BM}=k_{f}\left[1+\frac{\frac{v_{f}}{v_{a}}-1}{1+\frac{k_{a}}{k_{f}}\left(1+Z\left(\frac{v_{f}}{v_{a}}-1\right)/\left(\frac{v_{f}}{v_{a}}+1\right)\right)}\right]$$

The term *Z* is the fraction of fiber orientation to the macroscopic heat flow (*Z* = 1 when fibers are aligned perpendicular to the heat flux, *Z* = 0.66 when randomly arranged, and Z = 0.83 when arranged parallel to the heat flux). One critical orientation angle, $\psi =\cos^{-1}2/3\;\approx \;48.19{}^{\circ}$, was suggested in Stark and Fricke’s work [15]. Fibers with an orientation θ<ψ are considered parallel to the direction of heat flow, and fibers with θ>ψ are perpendicular. The final Stark-Fricke model is represented as:

(2) $$k=\left(i+1\right){\left[\frac{i}{k^{BM}}+\frac{j+1}{j}\frac{1}{k_{a}+\frac{4k_{f}Cd}{j\pi a_{ct}}}\right]}^{-1}$$

where $a_{ct}$ is the contact radius, and the *i* and *j* given as a function of the fiber orientation, are

(3) $$i=\frac{1}{2}s\cos\theta -1$$

(4) $$j=s^{2/3}\frac{{\left(0.5\sin\theta \right)}^{1/3}}{\pi {\left(1.5\left(1-\mu_{0}^{2}\right)p_{ext}/E\right)}^{2/3}}-1$$

where *s*, the geometrical parameter, is

(5) $$s={\left(\frac{\pi \rho_{f}/\rho }{0.5sin^{2}\theta \cos\theta }\right)}^{1/2}$$

where C is the connection parameter with a suggested value of 0.611; d is the fiber diameter; $a_{ct}$ is the contact radius; $\mu_{0}$ is the Poisson’s number of the fibrous material; $p_{ext}$ is the external pressure; E is the Young’s modulus; $\rho_{f}$ is the bulk density of fiber; and *ρ* is the bulk density of fibrous material.

## 3. Materials and Methods

### 3.1. Materials

**Figure 3:**
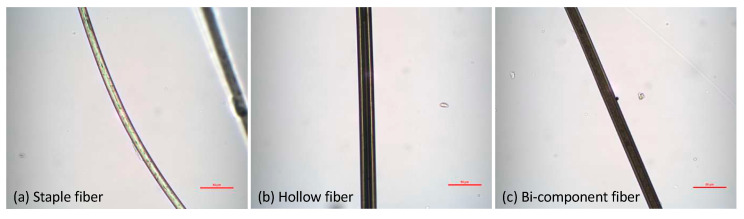
Longitudinal images of fibers: (**a**) staple fiber; (**b)** hollow fiber; (**c**) bi-component fiber.

The polyester nonwoven fibrous material was made of 45 wt.% staple polyester, 30 wt.% hollow polyester, and 25 wt.% bi-component polyester. Low-melting polyester fiber consists of the sheath part of a bi-component fiber, which is used to thermally bond the nonwoven structure. The preparation procedure of polyester nonwoven samples was reported in our previous work [32]. The longitudinal images of three types of polyester fibers are shown in Figure 3. The longitudinal images were captured at the Technical University of Liberec using the JENAPOL microscope (Jena, Germany) and NIS-elements software (AR 4.30.02 64-bit). Later, fifty fibers were measured for each type of fiber to ensure an accurate value. The mean diameters of the staple, hollow, and bi-component fibers were 13.19 ± 0.57, 24.45 ± 2.56, and 17.94 ± 0.82 µm, respectively. 

**Figure 4:**
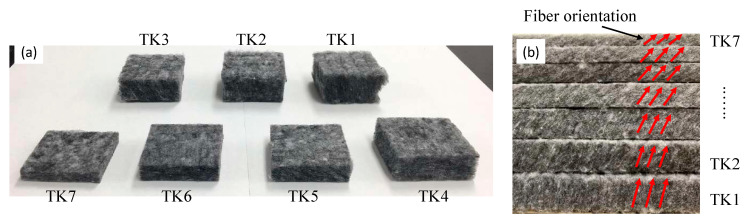
Polyester nonwoven samples (**a**) and cross-sectional macroscopic images (**b**).

**Figure 5:**
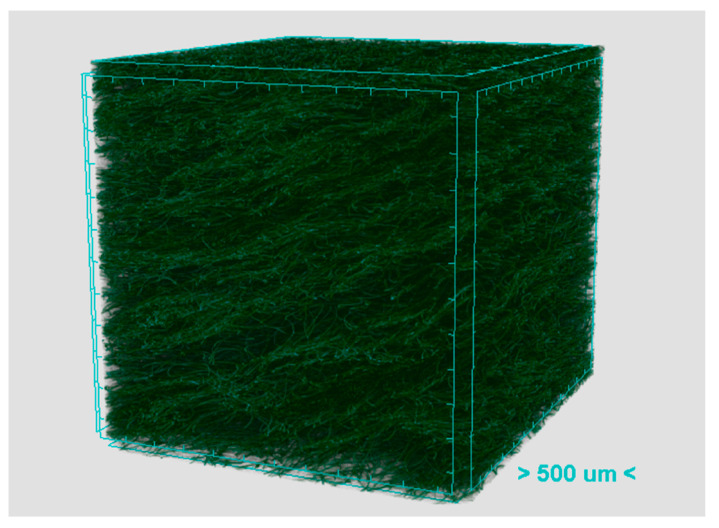
Tomography image of polyester nonwoven sample TK7.

The polyester nonwoven samples and their cross-sectional macroscopic images are illustrated in Figure 4. It can be seen that the decrease in thickness resulted in an increase in the fiber orientation angle. One tomography image of sample TK7 is presented in Figure 5. The x-ray micro computerized tomography was performed at the Technical University of Liberec, Czech Republic on a Bruker SKYSCAN 1272 (Billerica, MA, USA). Software CTVOX 3.3 was used to reconstruct the tomography image.

The characterization of polyester nonwoven fibrous samples has been reported in our previous work, as listed in Table 3 [32]. From Figure 5, it can be seen that the fibers were orientated in the same direction and had a similar orientation angle. Thus, it was assumed that the fibers were generally orientated to the same direction with the same orientation angle. The mean fiber orientation angle was estimated via ImageJ software (version 1.51). The mean fiber diameter of the polyester fibers was calculated by the length-weighted average method, as defined in Equation (6).

(6) $$d=\sum d_{i}\frac{l_{i}}{\sum_{i=1}^{3}l_{i}}$$

where $d_{i}$ is the *i*-th fiber type diameter obtained from the average value of 50 fibers, and $l_{i}$ is the corresponding total fiber length in a unit volume of nonwoven fibrous material:

(7) $$l_{i}=\frac{W_{i}}{\pi {\left(d_{i}/2\right)}^{2}\rho_{i}}$$

where $W_{i}$ is the *i*-th fiber type total weight in a unit volume of nonwoven fibrous material and $\rho_{i}$ is the corresponding fiber density.

### 3.2. Theoretical Study 

The thermal conductivity of fibers is a critical parameter in the listed models. It is obliged to figure out the value of polyester fibers to carry out the theoretical study. However, it is complicated and difficult to measure single fiber thermal properties, so referring to values from the literature is a reasonable approach. Baxter [23] estimated the thermal conductivity of polyester fiber in 1946 by measuring the thermal conductivity of pads of packed fibers with the same density. Kawabata [33] developed a specific apparatus to measure the thermal properties of 14 types of fibers in the longitudinal and transverse directions [34]. Militký et. al. [26] proposed the thermal conductivity of a typical polyester fiber with 40% crystallinity. Some values of the thermal conductivity of polyester fibers are listed in Table 4. 

**Figure 6:**
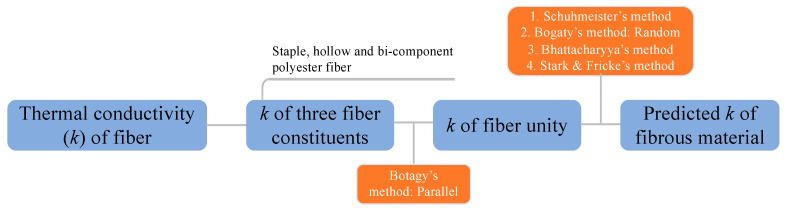
Theoretical study procedures according to fiber thermal conductivity.

The procedure for the theoretical study on thermal conductivity is illustrated in Figure 6. The fiber unity was defined as the entire solid phase (fibers) in the nonwoven fibrous material in the current study. Fiber thermal conductivities were used to estimate the values of three fiber constituents in the nonwoven samples. The conductivity of fiber unity was obtained via Botagy’s model for parallel arranged fibrous materials. Then, the thermal conductivity of the nonwoven samples was obtained by using the Schuhmeister, Bogaty, Bhattacharyya, and Stark-Fricke models. 

**Figure 7:**
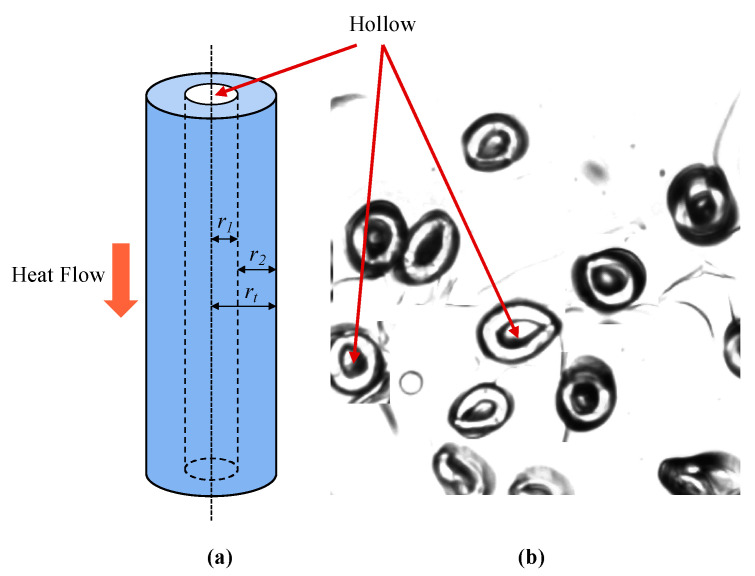
Heat transfer through the hollow fiber in the longitudinal (**a**) direction, and cross-sectional image of the hollow fiber (**b**).

Thermal conductivity of the hollow fiber should be evaluated since there was a 30 wt. % hollow fiber component in the samples. Heat transfer through the hollow fiber in the longitudinal direction is illustrated in Figure 7. The gas phase and solid phase were parallel arranged when heat transfer occurred in the longitudinal direction. The thermal conductivity under this situation can be simply estimated via model 5, as shown in Equations (8) and (9):

(8) $$k_{h-f}=\frac{v_{1}}{v_{t}}k_{1}+\frac{v_{2}}{v_{t}}k_{2}$$

where $v_{1}$, $v_{2}$, and $v_{t}$ are volumes of the gas phase (air), the solid phase, and the hollow fiber, respectively. It was assumed that the hollow continued in the hollow fiber. Then, the thermal conductivities of the hollow fiber can be represented as:

(9) $$k_{h-f}={\left(\frac{r_{1}}{r_{t}}\right)}^{2}k_{1}+\left(1-{\left(\frac{r_{1}}{r_{t}}\right)}^{2}\right)k_{2}$$

where $r_{1}$ and $r_{t}$ are the radius of the gas phase (air) and the hollow fiber, respectively; and $r_{2}$ is the thickness of the solid-phase wall in hollow fiber. The ratio between $r_{1}$ and $r_{t}$ was calculated based on the measured values via ImageJ software on the cross-sectional images of the hollow fiber (see in Figure 7). The ratio ($r_{1}/r_{t}$) was 0.433 with a standard deviation of 0.0345 by measuring 50 fibers.

The thermal conductivity of the fiber unity in the nonwoven fibrous materials was estimated from the thermal conductivities of the three fiber constituents by using a series arrangement model based on fiber volume fractions of each constituent when the solid and gas phases were in a parallel arrangement [35]. Then, the thermal conductivity of the fiber unity can be obtained as:

(10) $$k=v_{s-f}k_{s-f}+v_{h-f}k_{h-f}+v_{b-f}k_{b-f}$$

where $v_{s-f}$, $v_{h-f}$, and $v_{b-f}$ are the volume fractions of staple, hollow, and bi-component polyester fiber, respectively, and $k_{s-f}$, $k_{h-f}$, and $k_{b-f}$ are the thermal conductivities of staple, hollow, and bi-component polyester fibers, respectively. The thermal conductivity of the bi-component polyester fiber was assumed to be the same as the staple polyester fiber. In another situation where the two phases are perpendicularly arranged, the thermal conductivity of the fiber unity can be estimated by:

(11) $$k={\left(\frac{v_{s-f}}{k_{s-f}}+\frac{v_{h-f}}{k_{h-f}}+\frac{v_{b-f}}{k_{b-f}}\right)}^{-1}$$

The thermal conductivities of the three fiber constituents and fiber unity are listed in Table 5. Some of the models require the fractions of fibers that are parallel or perpendicular to the heat flow direction. The values of fractions can be easily calculated via the trigonometric function (see Equations (12) and (13). The volume fractions of air and fiber and the fractions of fiber orientation are listed in Table 6. Next, the final results of nonwoven samples are calculated via the methods demonstrated in Figure 6.

(12) $$x=\frac{1}{\tan\theta +1}$$

(13) $$y=\frac{\tan\theta }{\tan\theta +1}$$

### 3.3. Experimental Study 

Two different measurement methods were utilized to test the thermal conductivities of nonwoven fibrous samples. First, the samples were tested on an Alambeta device (SENSORA, Liberec, Czech Republic) at the Technical University of Liberec, Czech Republic. The measuring head of the Alambeta contains a copper block that is electrically heated to approximately 32 °C to simulate the temperature of human skin. The lower part of the heated block is equipped with a direct heat flow sensor that measures the thermal drop between the surfaces of a very thin, non-metallic plate using a multiple differential micro-thermocouple [36,37].

**Figure 8:**
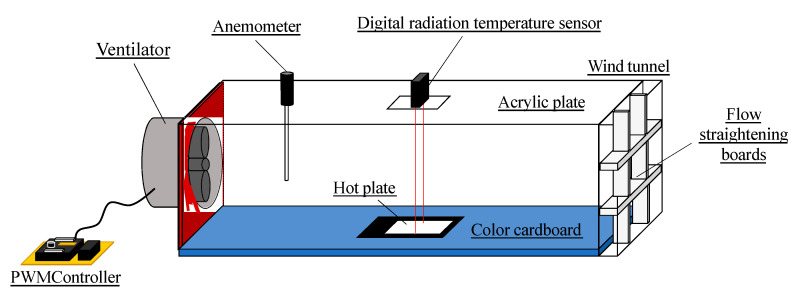
Sketch of the custom-built apparatus.

Furthermore, a new custom-built apparatus was used to measure the thermal conductivities at the Kyoto Institute of Technology [38]. The theory and procedure of thermal conductivity determination were adopted from the previous study (see [38]). As illustrated in Figure 8, the apparatus was composed of a hot plate (JUJI Field Inc. LABOPAD H, Tokyo, Japan), wind tunnel, a blower (SANYO DENKI Co. Ltd., 9SG 5724P5H61, Tokyo, Japan), a hot-wire anemometer (HARIO SCIENCE Co. Ltd., WGT-10, Tokyo, Japan), and a digital radiation temperature sensor (KEYENCE Corp. FT-H10, Osaka, Japan). Constant airflow was supplied to the wind tunnel by a blower, a specimen was placed on the heating portion of the hot plate, and the surface temperature of the specimen was measured by detecting the quantity of the infrared ray by using a digital radiation temperature sensor. Moreover, the temperature of the hot plate was set at 30, 50, and 70 °C, and the emissivity set for using the digital radiation sensor was unified to 1.0. 

## 4. Results and Discussion

In this section, the predicted thermal conductivities of nonwoven fibrous materials were first compared and analyzed. Then, the comparison between two different test methods was carried out. The validation of the models based on the measured values will be studied at the end, followed by one modified model. 

### 4.1. Predictions of Thermal Conductivity of Nonwoven Samples 

All of the modeling computations were processed in MATLAB_R2018b and the results are listed in Table 7. According to the four different values of fiber thermal conductivities, the values of nonwoven samples were listed in two groups. It can be seen that all of the predicted conductivities were less than 0.2 W m^−1^ K^−1^. 

**Figure 9:**
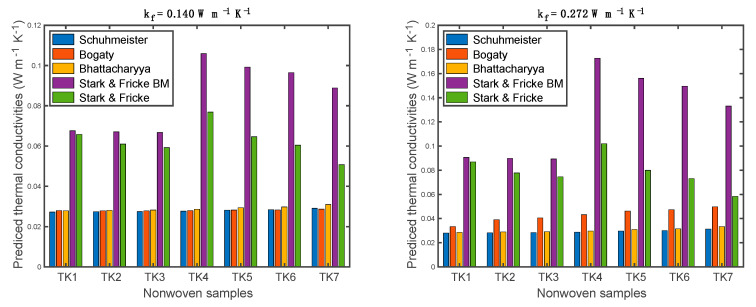
Predicted thermal conductivities of nonwoven samples based on different models.

The predicted thermal conductivities are shown in Figure 9. It is obvious that the results from Stark and Fricke exhibited much higher thermal conductivities when compared to other models. Meanwhile, results based on the Stark-Fricke model had a clear trend, which first slightly decreased, then reached the highest value, followed by a decrease. Results among the seven nonwoven samples based on the Schuhmeister and Bhattacharyya models had very small differences. When the fiber thermal conductivity was higher (i.e., 0.272 W m^−1^ K^−1^), the results based on the Bogaty model exhibited a clear increase. Generally, the increase in fiber thermal conductivity resulted in an increase in the thermal conductivity of the fibrous material. This can be confirmed in Figure 9. However, the increases in some predicted values (i.e., Schuhmeister and Bhattacharyya models) of nonwoven samples were relatively small, although the fiber thermal conductivity was nearly doubled. The reason behind this phenomenon can be the low fiber volume fraction (i.e., <0.06) when compared to the air volume fraction. Additionally, it is not easy to observe the effect of fiber orientation fractions on predicted thermal conductivity from Figure 9. Therefore, the effect of fiber volume fraction and fiber orientation fraction on thermal conductivity will be analyzed. 

**Figure 10:**
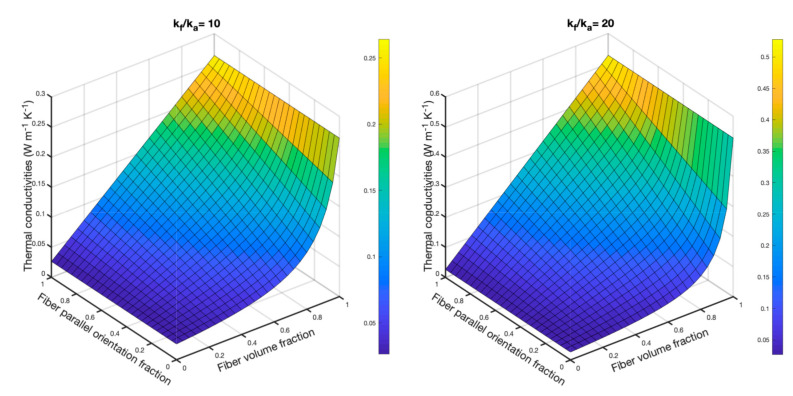
The effect of fiber orientation and fiber volume fractions on the thermal conductivity of nonwoven samples based on the Bogaty model.

The Bogaty model was selected since it involved both the fiber volume fraction and fiber orientation fraction and is widely used for theoretical study in literature. The influences of fiber volume and orientation fractions on the predicted thermal conductivity is shown in Figure 10. Thermal conductivities of conventional textile fibers are usually 5–20 times to the value of steady dry air [29]. The thermal conductivities of fibers with the values of 10 times and 20 times to air were adopted. The x-axis and fiber parallel orientation fraction represent the percentage of fibers parallel to the direction of heat flow. The fiber parallel orientation fraction ranged from 0 to 1, which shows that when the value is close to 1, the fibers are parallel to heat flux. In contrast, when it is low, the fibers are toward perpendicular to the direction of heat flow. It can be observed that the effect of fiber orientation on thermal conductivity was trivial when the fiber volume fraction was lower than 0.4. For fibers with higher thermal conductivity, fiber orientation had less influence when the fiber volume fraction was lower than 0.7. Meanwhile, the effect of fiber thermal conductivity on the fibrous material with a low fiber volume fraction was insignificant. For the nonwoven material with fiber thermal conductivity 10 times to air, the fiber orientation fraction exhibited significant influence when fiber volume fraction was around 0.7–0.9. Thus, it can be concluded that fiber volume fraction in the Bogaty model plays a more important role on the thermal conductivity of nonwoven samples compared to the fiber orientation. As shown in Table 3, the nonwoven samples had low fiber volume fractions with values from 0.0165 to 0.0533. Thus, it can explain why some models exhibited similar results although the fiber thermal conductivity increased to nearly 200%.

### 4.2. Measured Thermal Conductivity of Nonwoven Samples 

Measurements of the thermal conductivity of nonwoven samples were carried out on an Alambeta device and a custom-built device. The results of the thermal conductivities and their standard deviations are listed in Table 8. The Alambeta can rapidly test the thermal properties of fibrous materials by simulating the temperature of human skin [37]. However, since the upper and lower heat flow sensors are open and free during test, free convection and heat dissipation could occur at the edge of the specimen, especially for thick samples. The custom-built device was well designed. Based on the evaluation theory and procedure of the custom-built device [38], it only needed to measure the temperature of the specimen surface at three different given temperatures (30°, 50°, and 70°). Then, the least-squares method was used to obtain the thermal conductivity of the measured sample. 

**Figure 11:**
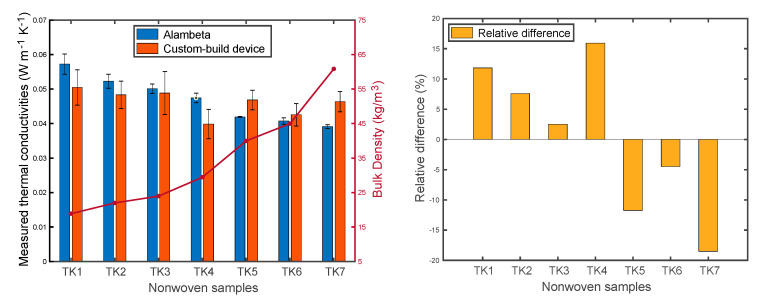
Comparison of the results from two different devices.

In order to compare these two measurement methods a formula, Equation (14), was applied to calculate the relative difference. The comparison and relative difference are shown in Figure 11. It can be found that as density increased, the results from the Alambeta slightly decreased, while the results from the custom-built device did not show a clear trend. Furthermore, the results from the Alambeta device exhibited relatively low standard deviation. Theoretically, the increase in density results in an increase in the fiber volume fraction, then the path of heat flow involves more fibers. Consequently, the thermal conductivity of fibrous materials will increase. Nevertheless, the measured thermal conductivities of nonwoven samples exhibited a contradictory or different phenomenon. This is due to the different fiber orientation among the nonwoven samples, as shown in Figure 4 and Figure 5. It has been stated that the fiber in the through-plane orientation has a critical effect on the thermal conductivity of fibrous material.

(14) $$\Delta =\frac{k_{Alam}-k_{Cust}}{k_{Alam}}\;100\%$$

where Δ is the relative difference and $k_{Alam}$ and $k_{Cust}$ are the results measured by Alambeta and the custom-built device, respectively.

In a comparison with the custom-built device, the Alambeta device showed higher results on samples with lower density (i.e., < 30 kg/m^3^), while the results were lower when the sample density was higher. The relative difference ranged from −18.50% to 15.92%. The biggest difference occurred for sample TK7 with a value of −18.5%. The most similar result was from sample TK4 with a 2.50% relative difference. Due to the uncertainties during measurement on the custom-build device, the effect of a different testing environment and operation procedure, a relative difference less than ± 20% was considered reasonable.

### 4.3. Validation of Thermal Conductivity Models

**Figure 12:**
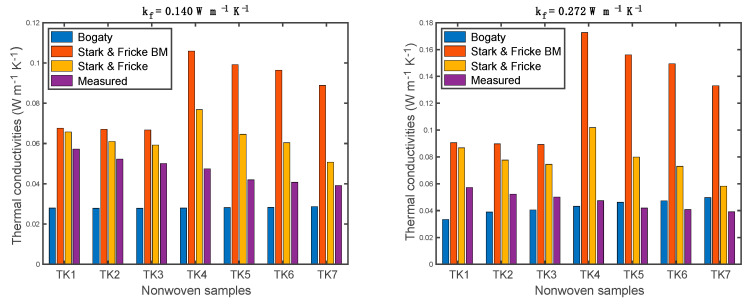
Predicted and measured thermal conductivities of the nonwoven samples.

Measured results from the Alambeta were selected to validate the thermal conductivity models. Since the Schuhmeister model is considered one empirical version of the Bogaty model and the Bhattacharyya model does not include the fiber orientation fraction, the results based on the Bogaty and Stark-Fricke models will be further compared with the measured values. The comparison between the predicted and measured thermal conductivities are shown in Figure 12. When the fiber thermal conductivity increased, the results based on the Stark-Fricke models exhibited a significant increase in samples TK4, TK5, TK6, and TK7. It can be seen that two models proposed by Stark et. al. overestimated the thermal conductivities of the nonwoven samples. The Stark-Fricke model exhibited the closest results to the measured values when the fiber thermal conductivity was 0.140 W m^−1^ K^−1^, while the Bogaty model showed the most similar results when the fiber thermal conductivity was 0.272 W m^−1^ K^−1^. An upsurge occurred in the predicted values based on the Stark-Fricke models. This can be explained by the fraction of fiber orientation, *Z*, changing from 0.83 to 1 in the Stark–Fricke models. By comparing all of the results, it is hard to conclude which model is the most suitable for polyester nonwoven fibrous materials.

### 4.4. Modification on Stark-Fricke Basic Model

As stated above, fiber thermal conductivity has an insignificant effect on the prediction of the Bogaty model when the fiber volume fraction is low (i.e., < 0.10), while it has a critical effect on the prediction based on the Stark-Fricke models. Thus, it is more reasonable to modify Stark-Fricke models for fibrous materials with different through-plane orientation and low fiber volume fraction. However, it is first necessary to confirm the fiber thermal conductivity. However, only one method was proposed by Kawabata to directly measure the fiber thermal conductivity [33,34]. Kawabata specifically measured thermal conductivities in both longitude and transverse directions. When heat flow transfers in fibrous material, it can transfer in varying directions instead of only in the longitude and transverse directions. Thus, the value of 0.140 W m^−1^ K^−1^ obtained by the estimation method was adopted for model modification [23]. Since the prediction process of the Stark-Fricke model is more complicated, only the Stark-Fricke BM (basic model) was modified. Moreover, Stark et. al. did not precisely describe the method to obtain fiber orientation factor (*Z*). The modification of the Stark-Fricke basic model was carried out via optimization on *Z*. The parameter inversion process was applied to optimize the fiber orientation factor. Assuming that the fiber and air thermal conductivities and their volume fractions are known, and *Z* is the independent variable, the thermal conductivity based on the Stark-Fricke basic model can be represented as k(Z). Consequently, the *Z* can be inverted by finding the minimum of the following equation:

(15) $$f\left(Z\right)=\left|k\left(Z\right)-k_{meas}\right|\to min$$

where $k_{meas}$ is the measured thermal conductivity. In this work, the minimization problem was solved via a Nelder–Mead optimization method [39]. The original and optimized *Z* are listed in Table 9.

**Figure 13:**
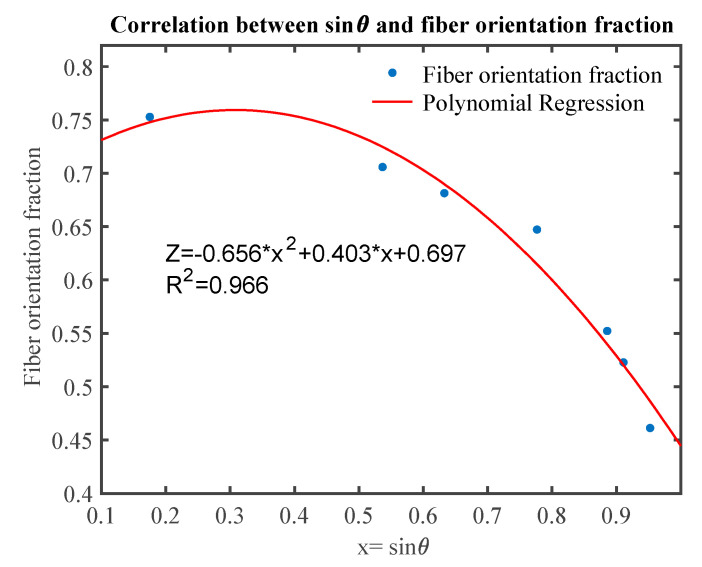
Predicted and measured thermal conductivities of nonwoven samples.

It is necessary to figure out the relation between fiber orientation angle (θ) and factor (*Z*). Two-degree polynomial fitting was applied to find this relation. The determination coefficient was 0.966, which means that sinθ and *Z* are strongly related based on the two-degree polynomial in Figure 13 Then, the fiber orientation factor *Z* can be represented as:

(16) $$Z=-0.656\;sin^{2}\theta +0.403\;sin\;\theta +0.697$$

**Figure 14:**
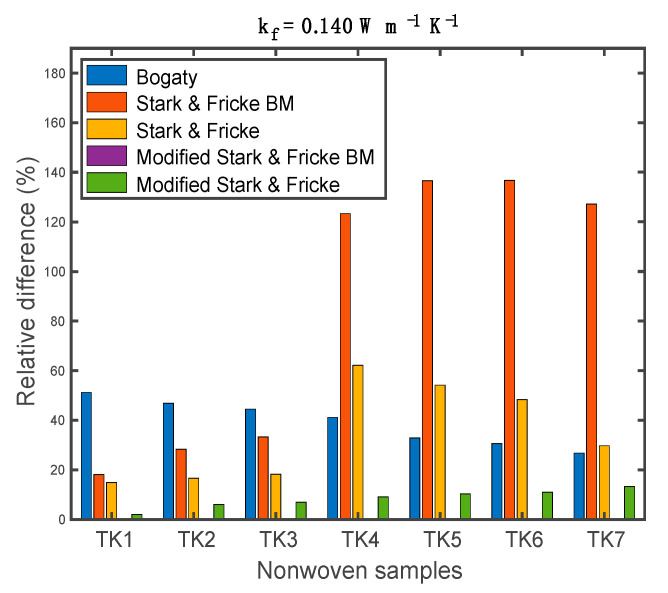
Predicted and measured thermal conductivities of the nonwoven samples.

In order to verify the modified Stark-Fricke basic model, the relative difference in the thermal conductivity of nonwoven samples was compared in Figure 14. The relative differences were computed as:

(17) $$\Delta =\frac{\left|k_{pred}-k_{Alam}\right|}{k_{Alam}}\;100\%$$

where $k_{pred}$ is the predicted thermal conductivity.

The Bogaty model exhibited a relatively low difference with a value less than 60%. The modified Stark-Fricke model presented a reasonable relative difference with a value ranging from 2% to 13.30%. Compared to the original Stark-Fricke basic model, the modified model showed a significant improvement in prediction accuracy. The maximum difference of the original Stark-Fricke basic model was nearly 140%, while the maximum value of the modified model was around 0.1%. For all of the samples, the relative differences of the modified Stark-Fricke basic model were 0.08%, 0, 0, 0.05%, 0.09%, 0.10%, and 0.05%, respectively. Thus, it can be concluded that the modified model can accurately predict the thermal conductivity of high-loft polyester nonwoven materials with different through-plane fiber orientation.

## 5. Conclusions

Estimations on the fiber unity of polyester in nonwoven fibrous materials were carried out based on referred fiber thermal conductivities. Several models such as the Schuhmeister, Bogaty, and Stark-Fricke models were selected to theoretically study the thermal conductivity of nonwoven samples with different fiber through-plane orientations and fiber volume fractions. The experimental investigations were completed on an Alambeta device and a custom-built device. Results of the theoretical and experimental studies were compared. A modification of the Stark-Fricke models was proposed. The following conclusions can be obtained from this work:

1. Predicted thermal conductivities of nonwoven samples exhibited big differences among the models. Changing of fiber thermal conductivity had a small effect on the results from the Schuhmeister and Bhattacharyya models. The Bogaty model was not suitable for nonwoven materials with a low fiber volume fraction (i.e., <0.1). The two models proposed by Stark et. al. showed much higher predictions compared to the other models.
2. Measured thermal conductivities from the Alambeta device and the custom-built device had varying relative differences between the samples. The value was between −18.50% and 15.92%, which is reasonable due to the measurement uncertainties, different environments, and other factors. Although sample density increased the thermal conductivity decreased. This is because the fiber orientation turns more perpendicular to the direction of heat flow when the sample density increased.
3. Two Stark-Fricke models overestimated the thermal conductivities of the nonwoven samples. The Bogaty model exhibited a relatively low difference with the values ranging from 26.73% to 51.21%. The original Stark-Fricke basic model showed a big relative difference (i.e., 18.13–127.18%). The modified model could accurately predict the thermal conductivities with a very small relative difference and can provide a reliable prediction of the thermal conductivity of polyester nonwoven fibrous materials.

## Figures and Tables

**Table 1:** Some semi-empirical models for thermal conductivity estimation.

No.	Thermal Conductivity	Reference
1	$k=\frac{1}{3}\left(v_{f}k_{f}+v_{a}k_{a}\right)+\frac{2}{3}\left(k_{f}k_{a}/\left(v_{a}k_{f}+v_{f}k_{a}\right)\right)$	Schuhmeister [22]
2	$k=0.21\left(v_{f}k_{f}+v_{a}k_{a}\right)+0.79\left(k_{f}k_{a}/\left(v_{a}k_{f}+v_{f}k_{a}\right)\right)$	Baxter [23]
3	$k=v_{f}^{m}k_{f}+k_{a}$	Verschoor and Greebler [24]
4	$k=k_{f}^{v_{f}}k_{a}^{v_{a}}$	Tavman [25]

**Table 2:** Some models for fibrous material with parallel, perpendicular, and random fiber orientations.

No.	Thermal Conductivity	Fiber Orientation to the Direction of Heat Flow	Reference
5	$k=v_{f}k_{f}+v_{a}k_{a}$	Parallel	Bogaty et al. [27]
6	$k=\frac{\;k_{f}k_{a}\;}{k_{a}v_{f\;}+k_{f}v_{a}}$	Perpendicular	Bogaty et al. [27]
7	$k=\left[1-\frac{1-k_{a}/k_{f}}{1+\frac{2\left(k_{a}/k_{f}\right)\left(v_{f}/v_{a}\right)}{\left(1+k_{a}/k_{f}\right)}}\right]k_{f}$		Bhattacharyya [16]
8	$k=x\left(v_{f}k_{f}+v_{a}k_{a}\right)+y{\left(\frac{v_{f}}{k_{f}}+\frac{v_{a}}{k_{a}}\right)}^{-1}$	Random	Bogaty et al. [27]
9	$k=\left[1-\frac{1-k_{a}/k_{f}}{1+\frac{\left(1+5\left(k_{a}/k_{f}\right)\right)\left(v_{f}/v_{a}\right)}{3\left(1+k_{a}/k_{f}\right)}}\right]k_{f}$		Bhattacharyya [16]

**Table 3:** Basic characteristics of the polyester nonwoven fibrous samples.

Samples Code	Thickness (mm)	Bulk Density (kg/m^3^)	Porosity (%)	Mean Fiber Orientation Angle (°)
TK1	26.93	18.85	98.350	10.09
TK2	23.08	21.99	98.074	32.46
TK3	21.18	23.96	97.901	39.26
TK4	17.23	29.46	97.420	50.96
TK5	12.7	39.96	96.500	62.36
TK6	11.28	44.99	96.060	65.65
TK7	8.34	60.85	94.671	72.25

**Table 4:** Thermal conductivity of polyester fibers in the literature.

$k_{f}\;(W\;m^{-1}\;K^{-1})$	Reference
0.140	Baxter [23]
0.272	Militký et.al. [26]
0.260	Stark et.al. [15]

**Table 5:** Thermal conductivities of three fiber constituents and fiber unity.

Referred Values [23,26] (W m^−1^ K^−1^)	Staple and Bi-Component Fibers (W m^−1^ K^−1^)	Hollow Fiber (W m^−1^ K^−1^)	Fiber Unity (W m^−1^ K^−1^)	Bogaty Models [27]
0.140	0.140	0.1187	0.1324	Parallel
0.272	0.272	0.2261	0.2556	Parallel

**Table 6:** Volume fractions of air and fiber, and the fractions of fiber orientation to the heat flow.

	TK1	TK2	TK3	TK4	TK5	TK6	TK7
$v_{a}$	0.9835	0.9807	0.9790	0.9742	0.9650	0.9606	0.9467
$v_{f}$	0.0165	0.0193	0.0210	0.0258	0.0350	0.0394	0.0533
x	0.8489	0.6112	0.5503	0.4478	0.3439	0.3116	0.2425
y	0.1511	0.3888	0.4497	0.5522	0.6561	0.6884	0.7575

**Table 7:** Predicted thermal conductivities of nonwoven samples based on different $k_{f}$.

$\mathrm{Referred}\;k_{f}(W\;m^{-1}\;K^{-1})$	Models	Predicted Thermal Conductivity of Nonwovens (W m^−1^ K^−1^)						
		TK1	TK2	TK3	TK4	TK5	TK6	TK7
$k_{f}$:0.140	Schuhmeister	0.0272	0.0274	0.0274	0.0277	0.0281	0.0284	0.0291
	Bogaty	0.0279	0.0278	0.0278	0.0279	0.0282	0.0283	0.0287
	Bhattacharyya	0.0278	0.0280	0.0282	0.0286	0.0294	0.0298	0.0310
	Stark & Fricke BM	0.0676	0.0671	0.0667	0.1059	0.0992	0.0964	0.0889
	Stark & Fricke	0.0658	0.0610	0.0592	0.0769	0.0646	0.0604	0.0507
$k_{f}$:0.272	Schuhmeister	0.0279	0.0282	0.0283	0.0288	0.0296	0.0301	0.0314
	Bogaty	0.0334	0.0390	0.0405	0.0432	0.0462	0.0473	0.0497
	Bhattacharyya	0.0285	0.0289	0.0291	0.0297	0.0309	0.0315	0.0334
	Stark & Fricke BM	0.0906	0.0898	0.0893	0.1727	0.1561	0.1495	0.1331
	Stark & Fricke	0.0868	0.0777	0.0745	0.1019	0.0799	0.0730	0.0583

**Table 8:** Measured thermal conductivities of the nonwoven samples.

Methods	Measured Thermal Conductivity of Nonwoven Samples (W m^−1^ K^−1^)						
	TK1	TK2	TK3	TK4	TK5	TK6	TK7
Alambeta	0.05726	0.0523	0.0501	0.04742	0.04194	0.04074	0.03912
SD	0.00293	0.00204	0.00135	0.00139	0.00013	0.00092	0.00053
Custom-build device	0.05049	0.04834	0.04885	0.03987	0.04686	0.04256	0.04636
SD	0.00512	0.00398	0.00623	0.00426	0.00284	0.00326	0.00294

**Table 9:** The original and optimized fiber orientation factor (*Z*).

	TK1	TK2	TK3	TK4	TK5	TK6	TK7
Fiber orientation angle (°)	10.09	32.49	39.26	50.96	62.34	62.34	72.25
Original Z	0.83	0.83	0.83	1	1	1	1
Optimized Z	0.7530	0.7059	0.6814	0.6473	0.5523	0.5228	0.4613

## Nomenclature

*h*	Material thickness (m)
*k*	Thermal conductivity (W m^−1^ K^−1^)
*k_a_*	Thermal conductivity of air (W m^−1^ K^−1^)
*k_f_*	Thermal conductivity of fiber (W m^−1^ K^−1^)
*k_s−f_*	Thermal conductivity of staple PET fiber (W m^−1^ K^−1^)
*k_h−f_*	Thermal conductivity of hollow PET fiber (W m^−1^ K^−1^)
*k_b−f_*	Thermal conductivity of bi-component PET fiber (W m^−1^ K^−1^)
*k_alam_*	Measured thermal conductivity on an Alambeta device (W m^−1^ K^−1^)
*k_Cust_*	Measured thermal conductivity on a custom-build device (W m^−1^ K^−1^)
*k_meas_*	Measured thermal conductivity (W m^−1^ K^−1^)
*k_pred_*	Predicted thermal conductivity (W m^−1^ K^−1^)
*l*	Fiber length (m)
*m*	Empirical coefficient
*R*	Thermal resistance (W m^−1^ K)
*v_a_*	Volume fraction of air
*v_f_*	Volume fraction of fiber
*x*	Fraction of fibers parallel to heat flow direction (x+y=1)
*y*	Fraction of fibers perpendicular to heat flow direction
*z*	Factor of fibers orientation to the heat flow direction
*δ*	Fiber in-plane orientation angle (°)
*θ*	Fiber through-plane orientation angle (°)
*ψ*	Critical fiber orientation angle (°)
*Δ*	Relative difference
